# Emerging Evidence for MicroRNAs as Regulators of Cancer Stem Cells

**DOI:** 10.3390/cancers3043957

**Published:** 2011-10-24

**Authors:** Aisha Sethi, Lynette M. Sholl

**Affiliations:** 1 Department of Pathology, Henry Ford Hospital, Detroit, MI 48202, USA; E-Mail: draishasethi@gmail.com; 2 Department of Pathology, Brigham and Women's Hospital and Harvard Medical School, Boston, MA 02115, USA

**Keywords:** microRNAs, cancer stem cells

## Abstract

Cancer stem cells are defined as a subpopulation of cells within a tumor that are capable of self-renewal and differentiation into the heterogeneous cell lineages that comprise the tumor. Many studies indicate that cancer stem cells may be responsible for treatment failure and relapse in cancer patients. The factors that regulate cancer stem cells are not well defined. MicroRNAs (miRNAs) are small non-coding RNAs that regulate translational repression and transcript degradation. miRNAs play a critical role in embryonic and inducible pluripotent stem cell regulation and emerging evidence supports their role in cancer stem cell evolution. To date, miRNAs have been shown to act either as tumor suppressor genes or oncogenes in driving critical gene expression pathways in cancer stem cells in a wide range of human malignancies, including hematopoietic and epithelial tumors and sarcomas. miRNAs involved in cancer stem cell regulation provide attractive, novel therapeutic targets for cancer treatment. This review attempts to summarize progress to date in defining the role of miRNAs in cancer stem cells.

## 1. Introduction

MicroRNAs (miRNAs) are a group of small (∼18–25 nucleotide) non-protein encoding RNAs that have recently been established as important regulators of gene expression, through which they play critical roles in cellular processes such as differentiation, proliferation and apoptosis. Numerous studies have demonstrated that the effects of miRNAs on gene expression have significant implications for development and oncogenesis. With their discovery in 1993 in *C. elegans* models [1,2], a new paradigm of gene regulation was born: miRNAs silence gene targets by binding to complementary regions in the 3′ untranslated region (UTR) of messenger RNAs (mRNA), leading to degradation of mRNA or inhibition of translation, ultimately causing a decrease in protein expression [3].

miRNAs appear to play an important role in the development and progression of cancers, a role that is mediated by alterations in the miRNA expression level or ability to bind its target. Aberrant miRNA expression or binding occurs through diverse mechanisms, including genomic amplification or deletion, epigenetic silencing, or point mutations occurring in the miRNA or target sequences [3], indicating that miRNAs can serve as either oncogenes (in which context they are referred to as “oncomirs”) or tumor suppressor genes. miRNAs have been implicated in tumor initiation [4], progression, metastasis [5] and, importantly, in the formation and maintenance of cancer stem cells [6].

Cancer stem cells (CSC) are defined as a group of cells within the tumor that have the capacity to self-renew and differentiate into multiple cell types [7,8]. As such, they have the potential to regenerate tumors after therapy and seed distant metastases; thus CSCs appear critical to development of treatment failure [9]. These observations are now driving a significant effort to identify therapeutic targets within the cancer stem cell population.

miRNAs have a demonstrated role in embryonic stem cell maintenance; similar features define embryonic and cancer stem cells. Particularly compelling evidence for a link between embryonic stem cells and cancer came with genome-wide expression analyses that revealed similar profiles in embryonic stem cells and aggressive forms of human cancer [10]. Given our increasing understanding of miRNAs as genome-wide regulators [11], manipulation of miRNAs holds potential as a means of attacking the cancer stem cell population [12].

Over 1,000 miRNA sequences have been recognized in the human genome [13]. In addition to holding promise as therapeutic targets, miRNAs are promising as biomarkers for diagnosis and prognosis. For instance, high levels of miR205a expression distinguishes primary lung squamous cell carcinoma from adenocarcinoma [14,15]. miR21 overexpression has been found to be a poor prognostic indicator in multiple tumor types including breast cancer [16] and pancreatic cancer, in which it predicts chemotherapy resistance [17,18] and tumor aggressiveness [19]. In contrast, reduced expression of miR146a is associated with increased pancreatic cancer invasion, with re-expression of miR146a leading to downregulation of IRAK11 mediated signaling through EGFR and NF-kappa B and reduction of cancer cell invasiveness [20]. *In vitro* studies demonstrated that application of a naturally occurring isoflavone to pancreatic cancer cells could increase miR146a levels and thus reduce invasiveness, thus providing a novel therapeutic target in this uniformly deadly disease [20].

One of the challenges facing the field of cancer stem cell biology is defining the characteristics of this population in any individual tumor. The first models of CSC were demonstrated using leukemia cells, in which CD34+/CD38- cell fractions alone were able to recapitulate disease morphology when transplanted into immunocompromised mice [21,22] In solid tumors, small populations of cancer stem cells have been defined by expression of cell-surface markers such as CD133 (in colon cancer) or CD44 (in breast cancer) and the ability to form tumors in immune-deficient mice [9,23-25]. Aldehyde dehydrogenase (ALDH) is highly active in stem cells in multiple tissue types [26-28], is detectable using enzymatic assays or by immunohistochemistry and may serve as a useful intracellular marker of this population [29]. The interplay between markers of cancer stem cell populations and miRNAs may have significant implications for targeting the stem cell niche for therapeutic purposes.

## 2. The Role of miRNAs in Embryonic Stem Cells and Induced Pluripotent Stem Cells

**Figure 1.**
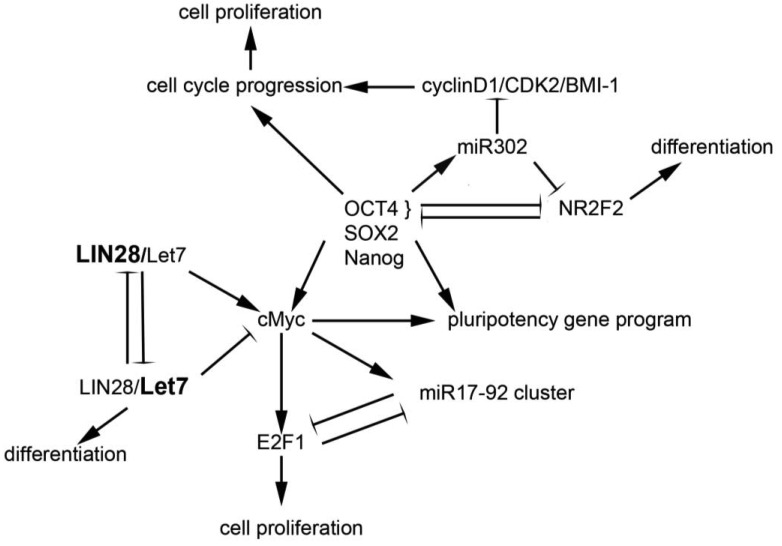
An overview of major gene and miRNA regulators of pluripotency and differentiation. LIN28 and let7 interact directly; LIN28 dominance tips the balance in favor of self-renewal whereas let7 dominance drives differentiation programs. Members of the miR17-92 cluster are turned on by cMyc and keep its pro-proliferative signals through E2F1 in check. Oct4 upregulates miR302; together they block differentiation signals via NR2F2; miR302 also puts the brake on the cell cycle, so cells in which it is highly expressed are relatively quiescent.

Drivers of pluripotency include the transcription factors Oct4, Nanog, Sox2, and cMyc, among others. The reprogramming of human embryonic fibroblasts and newborn and adult fibroblasts through the ectopic expression of human Oct4, Sox2, Klf4 and c-Myc generates human inducible pluripotent stem (iPS) cells [30,31]. These factors appear to engage in cross-talk with miRNAs, leading to coordinated regulation in embryonic stem cells; studies in these cells suggest that miRNAs are part of a core regulatory program that permits pluripotency, self-renewal, and differentiation [32]. A number of miRNAs have been identified as key regulators of pluripotency and/or self-renewal and proliferation; a few of these are discussed below and highlighted in Figure 1.

Evidence for the association between miRNAs and “stemness” mounted in studies showing that expression of OCT4, SOX2, NANOG, and LIN28 could reprogram adult human fibroblasts to inducible pluripotent stem cells [33], suggesting that the miRNA LIN28 and its regulatory target, let-7, play a critical role in maintenance of stem cells. Subsequent work demonstrated that the LIN28/let7 pair appears to act as a “toggle switch” between pluripotency and differentiation in embryonic stem cells [34]. The let7 family of miRNAs are maintained in their precursor forms by the binding of LIN28 to their terminal loops, thus blocking their processing to the mature state [35]. As a result, in conditions of high LIN28 expression, such as in stem cells, let-7 miRNAs are absent or expressed only at low levels [36]. Conversely, LIN28 is down-regulated during differentiation, relieving the inhibition on let7 [37]. Let7 miRNAs have been demonstrated to repress a broad range of targets, including Lin28, cMyc, nMyc, Sall4, and downstream effectors of the pluripotency genes NANOG, OCT4 and SOX2 [38]. These targets are critical elements of embryonic stem cell self-renewal programs, and their repression is required for differentiation. Thus, it appears that reduced LIN28 and increased let7 expression promotes a transition from pluripotency to tissue-specific programming [38]. Conversely, regulation of LIN28 and let7g by Oct4/Sox2/Nanog/Tcf3 in ES cells [37] promotes self-renewal and pluripotency. In sum, the interplay between LIN28 and let7 carefully regulates the balance between stemness and differentiation [39].

The miR17-92 cluster appears to be critical in both development and tumorigenesis in hematopoietic and solid organs. This polycistronic gene cluster, located on a region of ch. 13q13 that frequently undergoes copy number changes in human cancers, contains six highly conserved miRNAs that interact with apoptotic and cell cycle pathways [40]. In addition, the miR17-92 cluster is highly represented in expression profiles from embryonic stem cell populations, in which it appears to promote pluripotency and self-renewal [32]. *C-MYC*, one of the critical pluripotency genes in stem cells, as well as an established oncogene, promotes the expression of the miR17-92 cluster, which in turn downregulates E2F1 expression, whose accumulation drives the G1 to S phase transition in the cell cycle [41]. The E2F factors also bind the miR17-92 promoter, suggesting the presence of an autoregulatory loop [42]. Thus, the miR17-92 cluster appears to regulate the effects of c-myc on cellular proliferation via its interaction with the E2F factors; as such, and possibly as a function of the cell type and environment, the miR17-92 cluster can either suppress or permit the pro-proliferative effects of c-myc. Evidence that this cluster has oncogenic or tumor suppressor properties is supported by cytogenetic studies demonstrating frequent amplification [40] or loss of heterozygosity [43,44] at the 13q31 locus in different tumor types.

The miR302 family targets numerous genes important in early human embryogenesis [45,46], and is co-expressed with other ES cell genes such as Oct3/4, Sox2 and Nanog. Transfection of miR302 into cancer cell lines suppresses the expression of genes important in development and differentiation and appears to re-program differentiated cancer cells into a pluripotent ES-like state [45]. These miR302 reprogrammed cancer cells are relatively quiescent, as compared to other iPS cells. Via inhibition of cyclin D1/D2, CDK2, and BMI-1 expression, miR302 blocks G1-S cell cycle transition, leading to a low proliferation rate [47]. miR302 expression is induced by Oct4; together, Oct4 and miR302 silence the transcription factor NR2F2. Decreased levels of Oct4 and miR302 permit NR2F2 expression. In a positive feedback loop, NR2F2 represses Oct4 and regulates neural specification genes in early differentiation states [48]. Interestingly, in glioma-initiating cells, expression of the miR302-367 cluster of miRNAs occurs in tandem with suppression of Oct4 and Nanog and triggers a loss of self-renewal [49], findings which suggest disparate mechanisms of action in different cellular contexts.

## 3. Role of miRNA in Cancer Stem Cells

Cancer stem cells are broadly defined as a population that exhibits stem-cell specific cell surface or intracellular markers, forms spherules, and forms tumors when implanted into a receptive animal model. There is growing understanding of the regulatory networks that drive acquisition of the cancer stem cell phenotype and the role of miRNAs in these networks. Several organ-specific examples follow.

### 3.1. miR200 Family and Breast Carcinoma

Epigenetic regulation is key both in embryonic stem cells and in cancer. In ES cells, stemness is maintained by polycomb group (PcG) protein-mediated organization of promoter chromatin. Acting on CpG islands within the promoters of lineage commitment genes, PcG proteins facilitate normal development via histone modifications that allow a balanced but dynamic gene regulatory state [50]. Recent evidence suggests that similar regulatory elements act in tumorigenesis, with PcG family members recruiting DNA methyltransferases to CpG islands and leading to stable silencing of tumor suppressor genes via DNA hypermethylation [51]. These methylation states modulate, and are modulated by, miRNAs. miRNAs located within CpG islands can be epigenetically silenced, an important event in tumorigenesis when the miRNA targets an oncogene (as is the case for the miR127-BCL6 pair) [52]. Expression of the miR29 family of miRNAs binds and silences two DNA methyltransferases that are commonly upregulated in lung cancer, leading to restored expression of epigenetically-silenced tumor suppressor genes [53].

In a model of Src oncogene-transformed breast epithelial cells, CSC formation is dependent upon downregulation of miR200 family members [24]. In this model, the CSC population is defined as a CD44-positive, mammosphere-forming subset of cells that can form tumors in immune-deficient mice. The authors of this study note that while downregulation of miRNAs such as let7 occurs in both neoplastic transformation and CSC formation, expression of the miR200 family members is specifically altered in CSCs, suggesting a central role for miR200 in formation of this population. The mechanism of action of miR200 in regulating CSC formation appears to be epigenetic. Namely, miR200 interacts with Suz12, a component of the PRC2 polycomb complex. During neoplastic transformation, PRC2 methylates histones leading to aberrant silencing of tumor suppressor genes. miR200 expression inhibits Suz12 action; silencing of miR200 leads to significantly increased Suz12 transcript and protein expression in the CSC population. Suz12 consequently binds multiple targets in the genome, leading to histone methylation and gene silencing of multiple targets including WNT1, Sox2, and CDH1 (E-cadherin). The effects of Suz12 on e-cadherin in particular may contribute to the invasive and metastatic potential of breast carcinoma, with high miR200, low Suz12 and high e-cadherin levels associated with primary tumors and low miR200, high Suz12, and low e-cadherin observed in metastatic tumors [24]. Overexpression of miR200 leads to CSC death, raising the possibility that miRNAs may be useful therapeutics, especially in combination with chemotherapeutic drugs better suited to kill the non-CSC tumor population.

### 3.2. MicroRNAs, Microvesicles, and Metastases

MicroRNAs are thought to contribute to priming a stromal niche for tumor cells; in the context of metastases, miRNA delivery via microparticles permits “horizontal communication” between tumor and recipient stroma, even before the tumor implants at that site [54]. Microvesicles are plasma-membrane-derived particles that may contain cell-type specific proteins, nucleic acids, and other bioactive substances; these are released into the extracellular environment, exerting local and, if captured by the blood stream, distant effects. As compared to normal tissues, tumors shed microvesicles into the circulation in large amounts. The microvesicle fraction in the serum is enriched for miRNAs, and several studies indicate that these miRNAs have angiogenic potential [55-57]. One study demonstrated a CD105-expressing cancer stem cell population that preferentially produces microvesicles rich in pro-angiogenic mRNAs and miRNAs that enhance tumor implantation in a mouse model of renal cell carcinoma metastases to the lung [57]. The profile of the RNAs within the microvesicles from the cancer stem cell fraction appears to be enriched in pro-angiogenic and pro-tumorigenic factors that may enable the cancer stem cells to coordinate the spread of tumor. The mRNAs and miRNAs are delivered by the microvesicles to recipient cells, where they can be translated into proteins [58,59] or potentially trigger epigenetic reprogramming.

### 3.3. Lin28B in Colon Adenocarcinoma

LIN28 and its homologue LIN28B are key regulators of stem cell pluripotency, as discussed above. It is demonstrably important in colonic development, and overexpression of LIN28B is associated with tumorigenesis in multiple organs. LIN28 and LIN28B overexpression is found in approximately 15% of human cancers and is associated with poor outcomes [60]. Recently, King *et al.* demonstrated that LIN28B overexpression in colon adenocarcinoma in particular is a poor prognostic indicator [61]. Activated protein C (APC) mutations are common in colon adenocarcinoma, leading to Wnt pathway upregulation. Targets of the Wnt pathway include c-myc, which in turn upregulates LIN28B expression [62]. Constitutive expression of LIN28B in colon cancer cell lines turns on colonic stem cell markers PROM1 and LGR5 and enhances the metastatic capacity of these cancer cells in a mouse model [61] The effects of LIN28B in this model appears to be driven in part by the enhanced invasiveness and motility of LIN28B-overexpressing tumor cells. In contrast to embryonic stem cells, where let7 and LIN28 co-regulate each others' expression, let7 fails to inhibit the effects of LIN28B, suggesting that LIN28B can act independently of Let7 in this colon cancer model [63].

Various markers have been proposed to define the stem cell component of colon adenocarcinoma, including CD44, CD133, and CD166 [64-66]. Of note, one study of miRNA expression in CD133+ HT29 colon cancer cells failed to identify significant differences in let7 or LIN28B expression in this population as compared to a CD133-population, despite other features of stemness that were unique to the CD133+ cells [67]. These findings suggest that miRNA expression profiles may vary depending on the type of tissue examined (*i.e.*, primary tumor *vs.* cell line), as well as for individual tumor subtypes. Further studies are needed to establish a link between the colon cancer stem cell population and dysregulated LIN28 activity.

### 3.4. Tobacco Carcinogens and Epigenetic Regulation of miRNAs in Lung Carcinoma

The carcinogens in tobacco smoke are recognized to induce epigenetic changes in the DNA of epithelial cells of the upper aerodigestive tract [68]. These carcinogens, including methylnitrosylurea and benzo(*a*)pyrenediol epoxide, damage DNA, triggering the recruitment of DNA methyltransferase and histone modification [69]. Enrichment of histone marks (reversible methylation) and DNA cytosine methylation leads to gene silencing and is thought to induce dedifferentiation and acquisition of stem cell characteristics [51]. Exposure of lung epithelial cell cultures to tobacco carcinogens leads to epigenetic silencing of miR200b, miR200c and miR205 and phenotypic epithelial to mesenchymal transition (EMT) [69]. Acquisition of stem cell markers (CD44, CD133, ALDH1) and spheroid formation occurs after prolonged exposure to carcinogens, however this model does not induce complete transformation. Re-expression of miR200 family members or miR205 only partially reverses the stem cell phenotype, an observation that highlights the complex deregulation that occurs in carcinogenesis.

### 3.5. miR34 in p53-Mutated Pancreatic Cancer Stem Cells

P53, a key cell cycle regulator and tumor suppressor gene, is commonly mutated and inactivated in aggressive malignancies such as pancreatic adenocarcinoma. Studies examining the effects of tumor suppressor genes on miRNAs have demonstrated that p53 activates the transcription of the miR34 family of miRNAs. In turn, miR34 also acts as a tumor suppressor gene, via inhibition of bcl2, notch1/2 and c-met, all central pro-survival and pro-proliferative molecules [70,71]. CD44+/CD133+ cancer stem cell populations in p53-mutated pancreatic cancer cell lines contain low levels of miR34 and high levels of bcl2 and notch1/2. Re-expression of miR34 in this population triggers a loss of the CSC population, as demonstrated by a marked reduction in the CD44+/CD133+ subset, as well as inhibition of spherule formation and *in vivo* tumor growth. Importantly, overexpression of miR34 in these cells restored their chemo- and radiotherapy sensitivity. These observations suggest that miR34 is an important negative regulator of cancer stem cell renewal and may hold promise as a form of molecular therapy in pancreatic cancer [72].

### 3.6. miRNAs in Glioblastoma and Medulloblastoma

The presence of a cancer stem cell or tumor “initiating” population is well accepted in the highly malignant brain tumor glioblastoma multiforme (GBM), and is defined as CD133+ cells with neural stem cell-like properties (*i.e*., capable of forming neurospheres *in vitro* and neural tumors in *in vivo* models) [73]. Selected miRNAs are differentially expressed in CD133+ and CD133-GBM cells; miR9/9* are highly expressed in the stem cell fraction, where they appear to act as oncogenes by inhibiting CAMTA1, a tumor suppressor gene that appears to regulate cell proliferation [74].

Medulloblastomas are thought to originate from a number of different cerebellar cell populations, including granular cell progenitors and possibly neuronal stem cells. Sonic hedgehog (Shh) pathway drives cerebellar cell proliferation in normal development; abnormal activation of the Shh pathway in neural stem cells (NSC) triggers the development of medulloblastoma in animal models [75]. Notch proteins are also thought to play an important role in medulloblastoma pathogenesis. There is conflicting evidence as to the interaction of Notch and Shh [76], however both pathways appear to converge on helix-loop-helix factor HES-1, a transcription factor that is critical to progenitor cell self-renewal. miR199-5b blocks HES-1 translation, driving neuronal differentiation. In medulloblastoma this effect is specific to the CD133+ population [77]. Other miRNAs that interact with these pathways include miR17-92 and miR34a. miR17-92 promotes medulloblastoma formation in cerebellar granular cell precursors with constitutive Shh activation [78]. miR34a expression is induced by p53, inhibiting growth and triggering apoptosis, and it acts as a tumor suppressor in medulloblastoma via its negative regulation of the Notch ligand Delta-like 1 (Dll1) [79]. de Antonellis and colleagues demonstrate that miR34a plays a central role in Dll1-Notch signaling, regulating Notch 1 and 2 differentially depending on cellular context to drive differentiation and trigger apoptosis. Using an adenoviral-delivery system, these authors demonstrate that miR34 can drive neural differentiation of tumor neurospheres and impair tumor growth in a mouse model of medulloblastoma.

## 4. miRNAs as Therapeutic Predictors and as Therapy

There is ample evidence to suggest that miRNA expression profiles can predict response or resistance to an array of chemotherapeutics. For instance, downregulation of miR200b, -194 and -212 and upregulation of miR192, -424 and -98 are associated with docetaxel resistance in non small cell lung carcinoma [80]. Patients with advanced ovarian cancer whose tumors had reduced levels of tumor let7i expression had a shorter progression free survival [81]. *In vitro*, let7i-low ovarian and breast cancer cells demonstrate cisplatin resistance, suggesting that let7i modulates the effects of chemotherapy and may serve as predictive biomarker in ovarian carcinoma [81].

Attempts at miRNA inhibition and replacement have yielded some promising results. Oligonucleotides engineered to block endogenous miRNAs (so-called antagomiRs) can be delivered intravenously to specifically reduce the expression of their target miRNA and alter downstream mRNAs [82]. Thus, it is technically feasible to silence oncogenic miRNAs, suggesting that this approach might serve as a novel cancer therapy. Conversely, Kota *et al.* administered miR26a in a mouse hepatocellular carcinoma model using an adenoviral vector. These authors argue that “replacement” of miRNAs that are lost in a tumor-specific manner but are otherwise highly expressed in normal tissues should be associated with decreased toxicity. In fact, their model demonstrated that systemic miR26a therapy blocked hepatocellular carcinoma cell cycle progression and induced tumor cell apoptosis with a minimum of side effects [83]. In a conceptually similar model, Trang *et al.* demonstrated the efficacy of let7 replacement in reducing tumor burden using an intranasal, adenovirus delivery system in mice with KRAS-mutated lung adenocarcinoma [84].

In general, the global effects of miRNAs on the expression of mRNAs involved in cell cycle, survival, oncogenic pathways, and differentiation suggests that miRNA therapeutics may exert powerful effects on tumor cells, in particular in targeting the cancer stem cell niche. Bao *et al.* examined the efficacy of a novel synthetic curcumin analogue in treating pancreatic carcinoma. This curcumin analogue suppresses miR21 leading to re-expression of the tumor suppressor PTEN, and is associated with tumor cell death; the authors demonstrated that this agent effectively killed the cancer stem cell population, suggesting this may be a promising approach to treating patients with gemcitabine-resistant pancreatic cancer [85].

Dissecting the miRNA/mRNA pathways responsible for enabling local and distant tumor progression may uncover novel therapeutic targets. The miR200 family of miRNAs appears to play a central role in tumor invasion and metastasis via their negative regulation of ZEB1 and ZEB2, zinc finger proteins that act as transcriptional repressors of e-cadherin [86,87]. miR200 family members regulate the epithelial-mesenchymal transition (EMT), via mediation of growth factor signaling pathways including transforming growth factor-beta and platelet-derived growth factor D [88].

Overexpression of one miR200 family member, miR429, has been shown to reverse EMT in ovarian cancer cell lines [89]. Given the feasibility of miRNA adenoviral delivery, these observations raise the possibility that miR200 “replacement”, could serve as a novel metastasis-directed therapy.

## 5. Conclusions

The discovery of miRNAs has led to significant advances in our understanding of the normal and pathologic cellular regulatory systems. Much remains to be learned about cancer stem cells and their genetic and epigenetic regulation. However, given that many miRNA species serve as master regulators both in organism development and disease, they offer promising new targets in diagnosing and treating cancer, particularly in targeting the cancer stem cell population.
